# Human Papilloma Virus (HPV)-Mediated Oropharyngeal Squamous Cell Carcinoma Incidence Relative to Geographic Rates of HPV Vaccination in the United States

**DOI:** 10.7759/cureus.81459

**Published:** 2025-03-30

**Authors:** Delaney S Clark, Nhu Nguyen, Orly Coblens, Viran J Ranasinghe, Sepehr Shabani

**Affiliations:** 1 Otolaryngology - Head and Neck Surgery, University of Texas Medical Branch, Galveston, USA

**Keywords:** cancer, head and neck, hpv, otolaryngology, squamous cell carcinoma

## Abstract

Objective

This study aims to analyze the trends in the incidence of human papilloma virus (HPV)-mediated oropharyngeal squamous cell carcinoma (OPSCC) in relation to geographic rates of HPV vaccination in the United States.

Methods

The US Cancer Statistics (USCS) database and the National Cancer Institute’s (NCI’s) Surveillance, Epidemiology and End Results (SEER) program were used for incidence and population data collection; the Centers for Disease Control and Prevention’s (CDC) Teen National Immunization Survey provided vaccination data. Incidence of HPV-mediated OPSCC and the HPV vaccination rate were compared and analyzed using t-tests, ANOVA analyses, and linear regression analyses.

Results

Statistically significant differences between regions of the United States were observed when incidence and vaccination rates of OPSCC were analyzed. When incidence as a function of vaccination rate was analyzed, no significance was noted. Each region had an increase in the OPSCC incidence in the post-vaccine era compared to the pre-vaccine era.

Conclusion

We cannot conclude that any variance in OPSCC by region is due to HPV vaccination at this time. Because some regions show increased vaccination rates compared to others, it is likely that they will reach herd immunity first and be the first to see a decline in cases as the population ages. Because of the currently insignificant relationship between the vaccination rate over time and incidence rates, additional longitudinal analyses and cohort follow-up studies are needed to further assess the vaccine's impact.

## Introduction

Human papillomavirus (HPV) is the most common sexually transmitted infection in both the United States and around the world [1]. It was estimated that as of 2018, HPV has a prevalence of 40% among men and women aged 15-59 years in the United States [2]. According to the Centers for Disease Control and Prevention (CDC) data, there were an estimated 47,199 new cases of HPV-associated cancers between the years 2015 and 2019 [3]. HPV was first linked to cervical cancer in the 1980s, and its oncogenicity has been widely proven since [4]. As a result, the HPV vaccines were developed. Initially, the vaccines were created to prevent cancers of the reproductive system including the cervix as well as the anal passage [4,5].

However, at around the same time, the link between HPV infection and head and neck cancer was also noted with association with the oral cavity, pharynx, larynx, nasal passages, sinuses, and salivary glands. Of those, oropharyngeal cancer (OPC) remains the most prevalent, with 70% of OPC cases being HPV-mediated [6]. According to the CDC’s estimated annual number of cancer cases attributable to HPV, OPC makes up 39% of all cases when compared to other types of cancer [3]. In fact, as of 2021, OPC has surpassed cervical cancer as the most common HPV-mediated cancer in the United States, with 19,775 yearly cases as compared to 12,143 yearly cases of cervical cancer [7]. Despite the high relevance of HPV-mediated OPC, no formal studies have been conducted to determine the direct role of HPV vaccines in preventing OPC since the vaccine’s recommendation in 2006.

In the United States, the Gardasil-9 (9vHPV) is the only HPV vaccine currently being distributed. This vaccine protects against HPV types 6, 11, 16, 18, 31, 33, 45, 52, and 58 [8]. Of those, the HPV16 seropositivity is closely linked with OPC, with various studies finding a prevalence of 80 to 90% in OPC [9,10]. More notably, as the development of OPC is due to a persistent HPV infection, a 7-year follow-up study of patients with HPV-mediated OPC found that HPV16 infections were more likely to persist than others with 32% of HPV16 OPC cases persisting for at least five years with increasing viral load [11]. Interestingly, while OPC is linked to various lifestyle factors such as smoking, alcohol consumption, etc., studies have shown an association between HPV16 and OPC patients who are nondrinkers and nonsmokers [12].

With these factors in mind, this study aims to analyze the trends in the incidence of HPV-mediated OPC in relation to geographic rates of HPV vaccination in the United States using vaccination rate and incidence data. 

## Materials and methods

The US Cancer Statistics (USCS) 2001-2020 database and the National Cancer Institute’s (NCI’s) Surveillance, Epidemiology and End Results (SEER) program were used for data collection in this study. Incidence and population data were collected using the SEER*Stat Software program, version 8.4.3 in February of 2024. The combination of these two databases represents the entirety of the United States population from 2001-2020, excluding Puerto Rico. The 2001-2020 data set was the most recent one available for public use at the time. Because this study is not considered human subjects research, it did not require approval from the University of Texas Medical Branch Institutional Review Board.

Cases of oropharyngeal squamous cell carcinoma (OPSCC) were classified as potentially caused by HPV, based on the anatomic site of the cancer. These were identified by ICD-0-3 and are those listed here: base of tongue, lingual tonsil, overlapping lesion of tongue, soft palate, uvula, tonsillar fossa, tonsillar pillar, overlapping lesion of tonsil, tonsil, vallecula, anterior surface of epiglottis, lateral wall of oropharynx, posterior wall of oropharynx, branchial cleft, overlapping lesion of oropharynx, oropharynx, pharynx, Waldeyer’s ring, overlapping lesion of lip, oral cavity, and pharynx. Furthermore, ICD-0-3 codes were used to include histology codes 8050-8086 and 8120-8131 to include invasive cases. Additionally, only microscopically confirmed cases of OPSCC were included in the final counts.

We chose to sort data based on patient address at the time of diagnosis by year from 2001 to 2020, which resulted in a count of incidence of HPV-associated OPSCC by year, per state. Population data were then collected using the SEER*Stat software, age-adjusted to the US standard population in 2000, as this was the most recent data available for public use.

The CDC’s Teen National Immunization Survey from 2006-2020 was used to collect statewide data on the percentage of vaccination-aged patients who had received one or more doses of the HPV vaccine, stratified by partially or fully vaccinated patients. The vaccine was first approved by the FDA for women in 2006, followed by men in 2009. Because of this, there is no data published from 2006 and 2007 due to the low percentage of the population who were vaccinated at this time. The percentage of vaccinated individuals was first reported from 2008-2013 for just women; male data was gathered beginning in 2013, when greater than 50% of states had reported this data. Because of this, the percentage of vaccinated individuals in this project is reported as just females from 2008-2013, and as an average of male and female vaccination rates from 2014-2020.

The SEER*Stat software version 8.4.3 and Microsoft Excel version 16.85 (Microsoft Corporation, Redmond, USA) were used to conduct statistical analysis. Differences with p-values < 0.05 were considered significant. Incidence is reported as cases per 100,000. A combination of ANOVA single factor tests, t-tests, and linear regressions was used to perform statistical analysis.

To define geographic regions, guidelines from the United States Census were used to define regions as well as divisions. These are outlined in Table 1.

**Table 1:** Four regions and nine divisions of the United States as designated by the U.S. Census Bureau

Region 1: Northeast		
Division 1: New England	-	Division 2: Middle Atlantic
Connecticut	-	New Jersey
Maine		New York
Massachusetts		Pennsylvania
New Hampshire		
Rhode Island		
Vermont		
Region 2: Midwest		
Division 3: East North Central	-	Division 4: West North Central
Indiana	-	Iowa
Illinois		Kansas
Michigan		Minnesota
Ohio		Missouri
Wisconsin		Nebraska
		North Dakota
		South Dakota
Region 3: South		
Division 5: South Atlantic	Division 6: East South Central	Division 7: West South Central
Delaware	Alabama	Arkansas
District of Columbia	Kentucky	Louisiana
Florida	Mississippi	Oklahoma
Georgia	Tennessee	Texas
Maryland		
North Carolina		
South Carolina		
Virginia		
West Virginia		
Region 4: West		
Division 8: Mountain	-	Division 9: Pacific
Arizona	-	Alaska
Colorado		California
Idaho		Hawaii
New Mexico		Oregon
Montana		Washington
Utah		
Nevada		
Wyoming		

## Results

There were 328,565 cases of HPV-associated OPSCC diagnosed in our chosen age range during the years of 2001-2020 across all states. 134,508 (40.9%) of these cases occurred in the Southern region of the United States, 72,353 (22.1%) in the Midwest, 63,543 (19.3%) in the West, and 58,161 (17.7%) in the Northeast.

Incidence rates were averaged from 2006 to 2020 for each state and compared by region. The division with the greatest average incidence was the East South-Central division, with an average incidence of 9.02 cases per 100,000 persons. The division with the lowest average incidence over the same period is the Mountain division, with an average incidence of 6.65 cases per 100,000 persons. The difference between these two regions was statistically significant with a p-value of 0.0009. However, when the slope of the best-fitting line was analyzed with incidence as a function of time for each state, there was no significant difference noted between regions. Only one state, the District of Columbia from the South Atlantic division, was noted to have a negative slope at -0.15 cases/year. When we examined 2001-2006 data separately, the division with the highest average incidence was the South Atlantic at 6.47 cases/year, and the lowest was the Mountain division, at 4.67 cases/year. The difference between these two was also significant with a p-value of 0.002.

When examining variations in vaccination rates between regions and divisions, we looked at both the average vaccination rate from 2006-2020, as well as the change in the trend of vaccination in each state. When average vaccination rates were compared, it was noted that there was a significant difference between the division with the highest vaccination rates (New England, 53% vaccination) and lowest vaccination rates (East South, 32.79% vaccination) with a p-value of 0.0005. Vaccination rate and incidence averages between 2006 and 2020 by region can be further examined by looking at Table 2. When we examined trends in vaccination between divisions, there was no statistically significant variance noted.

**Table 2:** Average vaccination rate and incidence and trends in vaccination and incidence from 2006-2020 by region and division

		Average Vaccination Rate: 2006-2020	Trend in Vaccination: 2006-2020	Average Incidence Rate: 2006-2020	Trend in Incidence: 2006-2020
Region 1: Northeast		49.16	1.68	8.11	0.25
Division 1: New England		53.26	1.72	8.96	0.29
Division 2: Middle Atlantic		45.06	1.65	7.26	0.22
Region 2: Midwest		40.53	2.13	7.82	0.28
Division 3: East North Central		39.63	2.17	8.15	0.27
Division 4: West North Central		41.43	2.09	7.50	0.29
Region 3: South		36.72	1.75	8.58	0.22
Division 5: South Atlantic		41.31	2.11	8.57	0.22
Division 6: East South Central		32.79	1.55	9.03	0.22
Division 7: West South Central		36.06	1.59	8.16	0.23
Region 4: West		41.47	1.58	6.92	0.20
Division 8: Mountain		38.16	1.63	6.65	0.20
Divison 9: Pacific		44.78	1.53	7.20	0.20

**Figure 1:**
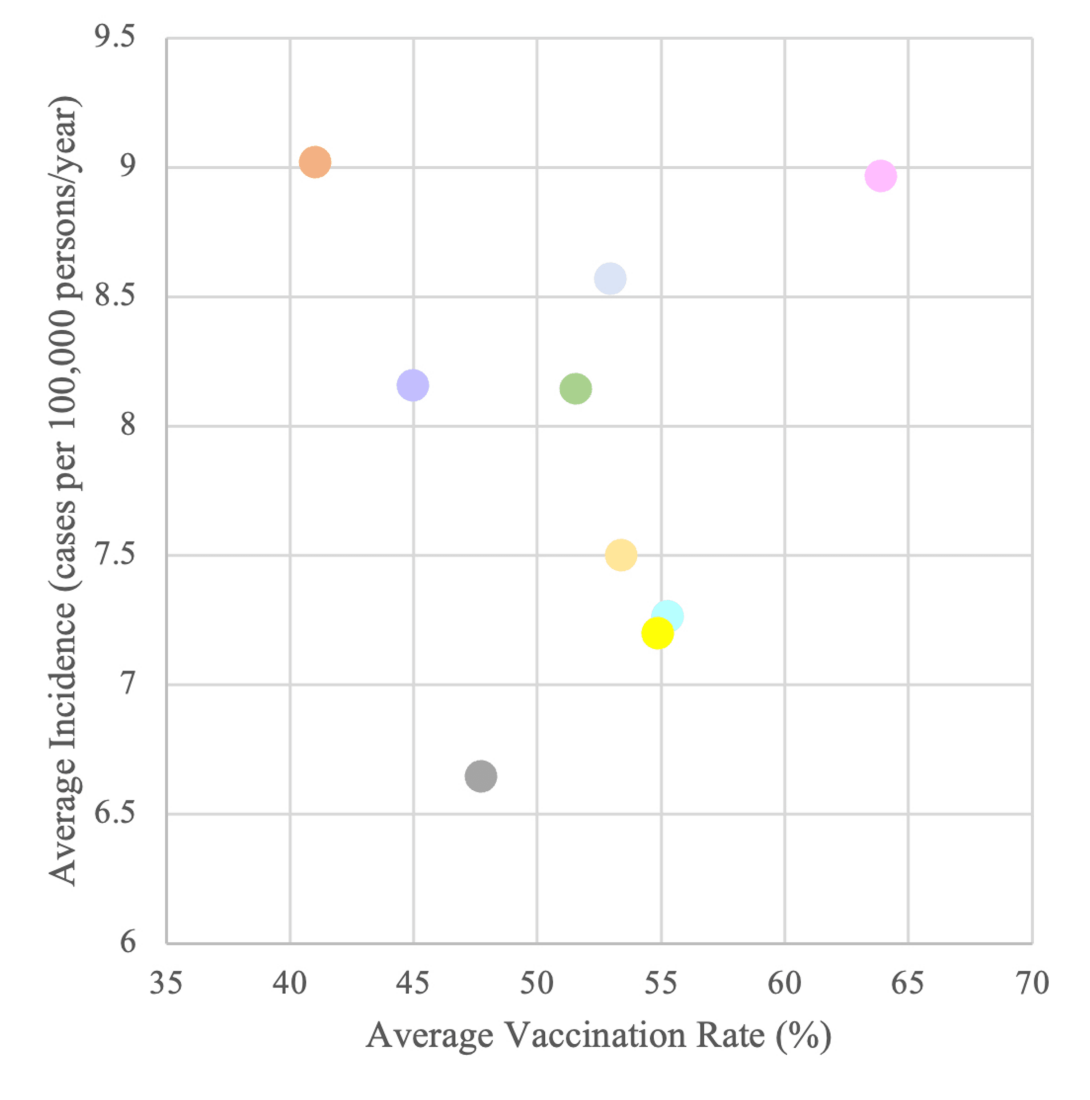
Division vaccination rate vs. incidence. Description: Plot of average vaccination rate (%) and average incidence (cases per 100,000 persons/year) from 2006 to 2020 by division Pink: New England; Teal: Middle Atlantic; Green: East North Central; Pale Yellow: West North Central; Blue: South Atlantic; Orange: East South Central; Purple: West South Central; Gray: Mountain; Yellow: Pacific

Furthermore, we wanted to examine the relationship between vaccination rates and incidence of OPSCC within states and divisions. A linear regression with vaccination rate over time as the X-variable and incidence rates over time as the Y-variable was completed with average values for each division. None of the regression analyses produced an output that was statistically significant, and none of them showed a negative relationship either, indicating that regions or divisions with higher vaccination rates do not necessarily have a decreasing incidence of OPSCC. The graph of average vaccination rate versus incidence for each division, as analyzed with linear regression, is plotted in Figure 1.

Lastly, to further determine if the HPV vaccination effects on OPSCC can be observed yet, we analyzed if there was a difference in the incidence of each region in the pre-vaccine and post-vaccine period. The results of this analysis are included in Table 3. Every division and region observed a significant increase in average incidence in the post-vaccine area compared to pre-vaccine, indicating that cases are still rising despite vaccination efforts. Additionally, the trend in incidence also increased in all divisions but three; in the three divisions with decreasing trends, none of these were statistically significant. These results indicate there is no current observable difference in the rate of increase of HPV-mediated OPSCC between regions of the United States.

**Table 3:** Average incidence and trends in incidence from 2001 to 2006 compared to 2006-2020 by region and division *indicates a statistically significant difference between time periods, p < 0.05

		Average Incidence: 2001-2006	Average Incidence: 2006-2020	Trend in incidence: 2001-2006	Trend in Incidence: 2006-2020
Region 1: Northeast		5.70	8.11	0.23	0.26
Division 1: New England		6.15*	8.96*	0.3	0.29
Division 2: Middle Atlantic		5.25*	7.26*	0.16	0.22
Region 2: Midwest		5.18	7.83	0.17	0.28
Division 3: East North Central		5.55*	8.15*	0.23	0.27
Division 4: West North Central		4.81*	7.50*	0.11*	0.29*
Region 3: South		6.16	8.58	0.19	0.22
Division 5: South Atlantic		6.47*	8.57*	0.14	0.22
Division 6: East South Central		6.18*	9.02*	0.31	0.22
Division 7: West South Central		5.84*	8.16*	0.12*	0.23*
Region 4: West		4.85	6.93	0.21	0.20
Division 8: Mountain		4.67*	6.65*	0.18	0.2
Divison 9: Pacific		5.03*	7.2*	0.24	0.2

## Discussion

This study demonstrated significant variances in the incidence of HPV-associated OPSCC between geographic regions, as well as rates of vaccination. Because linear regression analysis did not produce significant results when comparing rates of vaccination directly with incidence trends, it can be concluded that the differences in OPSCC observed between divisions are not a direct result of vaccination. This finding is consistent with previously published literature; there have been noticeable decreases in the incidence of HPV infection, but because OPSCC takes many years to develop, direct effects of the vaccine have yet to be observed [13]. The HPV vaccine was first introduced in 2006 to girls aged 11-12, while later expanding the recommendation to girls as young as 9 and catch-up vaccination through age 26 [14]. Because of this, the first women to have received the HPV vaccine are now around 29-30 years old, and the average age of HPV-mediated OPSCC patients at diagnoses is 56.9 years old, so it is consistent that we have not been able to observe any direct effects of vaccination thus far [15].

Additionally, because the recommendations and developments in HPV vaccine research are constantly being adapted to the needs of the public, some data collection was limited by this. For example, the first data reported for 2008 was the percentage of female patients within the selected age range who had received at least 1 dose of the Gardasil vaccine. In 2009, the data included women who had received one dose of the bivalent HPV vaccine, and in 2010, data included women who had received at least three doses (full vaccine series) of either the bivalent or quadrivalent HPV vaccine, leading to a decrease across all states for overall percentage vaccinated that year. When male data first became available in 2011, less than 50% of states were publishing this data, so we did not average male and female vaccination rates until greater than 50% of states were reporting, which was in 2013. Because of the nationwide lag in reporting male data, there was also a decrease in percentage vaccinated observed in 2013.

One major study limitation includes the Sars-CoV-2 pandemic in 2020 causing a delay in patients being diagnosed with various conditions, including OPSCC, as a pseudo-decrease or lack of incidence data was observed in greater than 50% of states that year. Additionally, the HPV vaccine was additionally recommended for exclusively female patients, and seeing as the majority of OPSCC cases are diagnosed in men, our study was not able to fully assess associations between percentage vaccinated and cases of OPSCC in real time. There were also variations in the type of vaccine recommended and the amount of vaccines considered a full dose, as referenced above, so this presents a limitation in data analysis as well. We believe the true effects of the HPV vaccine and its potential decrease in the incidence of HPV-associated OPSCC will be witnessed after the vaccine has been administered for another 20-30 years, given the gap between recommended vaccination age and average age at diagnosis.

Our data showed significant variances between regions and divisions when it comes to annual incidence of OPSCC and vaccination rates and an overall increase in incidence in the post-vaccine era compared to the pre-vaccine era, which are likely due to the delayed effects of the HPV vaccine. The South had the overall highest incidence of HPV-mediated OPSCC across the analyzed period and had the lowest rate of HPV vaccination but had the second lowest rate of increase in cases. It has been shown previously that areas with lower rates of vaccination have increased prevalence of HPV [16]. Paradoxically, the Northeast had the highest rates of vaccination, but the second highest average incidence observed. Zhetpisbayeva et al. described a phenomenon in which patients in rural areas are often seen to have lower rates of HPV vaccination, likely due to a myriad of factors including lack of public health education, socio-economic barriers, and geographic barriers [17]. The South houses almost half of the total rural population in the United States, which is consistent with the previously mentioned lower rates of HPV vaccination in rural areas [18]. In the coming years, areas with greater vaccine administration, such as the Northeast, will likely reach herd immunity first and will be the first to observe a decrease in the incidence of HPV-mediated OPSCC, but this will likely not be until the first people who received the vaccine reach the average age of OPSCC diagnosis.

## Conclusions

Overall, our data is consistent with studies that show we cannot yet observe decreases in the incidence of HPV-mediated OPSCC as a direct result of the HPV vaccine. However, we did see statistically significant variances in both average incidence and vaccination rates between different regions of the United States, potentially indicating that some regions will reach herd immunity sooner than others and may be first to see decreases in OPSCC incidence with time. This delay is likely due to the gap in recommended vaccination age and average age at diagnosis. Because of this, future studies should focus on regions and divisions with the highest and lowest vaccination rates to determine if different geographic areas are observing effects of the vaccine quicker than others. Additionally, studies within individual states should focus on comparing incidence and vaccination rates between rural and urban counties to further investigate effects of HPV vaccines, project which areas may see increases or decreases in OPSCC in the future and determine which counties to focus on for public health and HPV vaccine education. The inconsistency in incidence reporting affecting the relationship between incidence rate and vaccination could be elucidated in future studies with access to more extensive data on sociodemographic factors such as age stratifications and race. The increase in the incidence rate despite vaccination could also be linked to confounding factors such as improvements in diagnostic tests, potential HPV serotypes not covered by the vaccine, and changes in smoking habits such as the rise in popularity of electronic cigarettes.
